# Editorial: Evidence-Based Anti-Inflammatory, Anti-Hypoperfusion and Anti-Anxiety/Insomnia Therapies Show Promises for TBI-Induced Post-Traumatic Symptoms and Cognitive Deficits: Advances in Diagnosis and Treatment of TBI-Induced Neurodegeneration and Cognitive Deficits

**DOI:** 10.3389/fneur.2021.695629

**Published:** 2021-08-11

**Authors:** Guoqiang Xing, Yu Zhang, Yumin Zhang, Maheen Mausoof Adamson, Ansgar J. Furst, John Wesson Ashford

**Affiliations:** ^1^Affiliated Hospital and the Second Clinical Medical College of North Sichuan Medical University, Nanchong Central Hospital, Nanchong, China; ^2^Lotus Biotech.com LLC, Gaithersburg, MD, United States; ^3^School of Medicine, Stanford University, Stanford, CA, United States; ^4^Veterans Affairs Palo Alto Health Care System, Palo Alto, CA, United States; ^5^Uniformed Services University of the Health Sciences, Bethesda, MD, United States

**Keywords:** traumatic brain injury, post-traumatic symptoms, cognitive deficits, evidence-based therapies, innovative diagnosis

A progressive series of chronic microhemorrhages and inflammatory insults on the cerebrovascular system and neural-network integrity can lead to significant neurological and cognitive impairments following mild-to-moderate traumatic brain injury (mTBI). Evidence-based effective treatment is needed to stop this progression. Seventeen studies in this Research Topic (4 reviews, 2 prospective, 4 animal/cellular model studies, 5 clinical studies, and 2 clinical case reports) have investigated the mechanisms, diagnosis, and novel therapies of TBI-induced neuropathology and cognitive impairments. Activated inflammatory microglia, elevated Inflammatory cytokines (specifically CCL2 and IL-1β), oxidative stress, blood-brain barrier (BBB) disintegration, microvascular inflammatory injury-induced de-coupling between high metabolic demands and compromised cerebral blood flow (CBF)/perfusion, and brain network senescence/dysfunction associated with hyper-phosphorylated tau tangling appear to be the important pathological mechanisms following TBI. These disruptions of normal brain function underlie the disorders of consciousness, insomnia, depression, and neurological and cognitive impairments that follow mTBI. These pathological changes can now be determined by innovative assays and neuroimaging technologies. The advances in this field are leading to innovative treatments for the disorders which follow mTBI.

Developing approaches include intraneuroendoscopic removal of subdural hematomas, protecting BBB integrity by methylene blue or by medicinal herbs, controlling neuroinflammation by endocannabinoids, improving CBF/hypoperfusion by photobiomodulation or by executive function training (including computerized training and playing mahjong) or by transcranial direct current stimulation (tDCS), improving insomnia by cognitive-behavioral therapy. All of these approaches appear to be promising interventions to improve post-traumatic symptoms and cognitive deficits associated with mTBI.

As long-term repetitive sub-concussive impacts induce subtle neuromotor dysfunction in animals, similarly, clinicopathological changes and dementia in a 93-year-old street boxer are likely due to the brain concussions that occurred many years before. Accordingly, early diagnosis and treatment could mitigate the damage from repeated sub-concussive impact-induced chronic brain inflammation and resulting subjective cognitive decline (SCD) that had occurred in early life. Measures are developing which will likely provide effective prophylactic approaches that prevent dementia.

SCD, now recognized as an occasional preclinical sign of Alzheimer's disease (AD) and dementia, may be common in people with a history of TBI. Si et al. reviewed recent progress in SCD research and its relevance to TBI-induced cognitive deficits. SCD is characterized by early self-perceptive cognitive decline before the detection by objective tests. If identified, SCD can be treated/reversed more easily than later stages of progression from mild cognitive impairment (MCI) to dementia.

Sun et al. showed that serum CCL2 level was elevated over 3 months and was associated with the severity of post-concussion symptoms in 52 patients with acute mTBI compared to 54 healthy subjects, whereas elevated IL-1β was associated with worsened working memory in the acute phase of TBI. Thus, elevated inflammatory CCL2 and IL-1β could be predictors of post-concussion symptoms and cognitive outcomes of mTBI.

By studying inflammatory factors, Shang et al. showed that IL-1β effectively induced cellular senescence in rat astrocytes that was accompanied by increased total and phosphorylated tau. Similarly, both oligomerized amyloid β (Aβ) and H_2_O_2_ induced cellular senescence in rat astrocytes by NLRP3-mediated IL-1β secretion. They proposed that adverse stimuli-induced NLRP3 activation and IL-1β production are potentially diagnostic biomarkers and therapeutic targets for AD/dementia.

Shang et al. reported reduced serum miR-451 levels in patients with intracerebral hemorrhage (ICH) compared to healthy controls. They further evaluated the role of miR-144/451 cluster in ICH mice model. Deletion of miR-144/451 cluster exacerbated neurological deficits and brain edema, significantly promoted TNF-α and IL-1β secretion and oxidative stress in the perihematomal region of knockout mice compared with wild-type ICH animals, supporting a neuroprotective role of miR 144/451against ICH-induced inflammation and oxidative stress.

Yang et al. presented clinicopathological evidence of the coexistence of AD with hyper-phosphorylated tau-pathology, **c**hronic traumatic encephalopathy (CTE), dementia with Lewy bodies, and hippocampal sclerosis with TDP-43 pathology in a 93-year-old former street boxer with a pre-mortem diagnosis of severe dementia. CTE is a tauopathy in which multifocal perivascular phosphorylated tau aggregates accumulate in astrocytes, neurons, and neurites at the depths of the cortical sulci. These results suggest that early repetitive sub-concussive impacts may trigger pre-mortem pathologies of CTE, AD, and dementia.

Lavender et al. report that highly repetitive sub-concussive impact with a lightweight drop (25 g) onto anesthetized female rats for 12 weeks induced detectable/subtle neuromotor dysfunction i.e., increased foot slips in beam-walk and rotarod tests. Thus, long-term repetitive sub-concussive impacts may also be a mechanism underlying clinicopathological changes of the 93-year-old street boxer reported by Yang et al..

Tanaka et al. reviewed how modulation of the endocannabinoids (eCBs) system (composed of cannabinoid receptors, ligands, and metabolic/biosynthetic enzymes) could facilitate the homeostatic microglia to adopt either the (M1) state, which secretes mediators of the proinflammatory response, or to the (M2) state, which mediates the resolution of neuroinflammation and tissue repair/remodeling in neuropathological conditions. They propose that cannabinoid 2 receptor signaling pathway plays a critical role in shifting the microglia from the pro-neuroinflammatory (M1) state to the anti-inflammatory/neuroprotective (M2) state.

Du et al. showed in a non-randomized controlled trial that the intraneuroendoscopic technique (INET) is significantly better than traditional burr hole drainage (BHD) in the treatment of subacute and chronic septal subdural hematoma in terms of hematoma recurrence rate and subdural drainage tube (SDT) placement duration at 6-months post-operative follow-up.

Chronic microvascular injury/microbleeding following TBI may lead to inflammation and vascular cognitive impairment (VCI) which is difficult to diagnose. In their review paper, Wang et al. proposed MRI as the first choice for suspected VCI to evaluate brain atrophy, infarction, white matter hyperintensity, and hemorrhage. Resting-state functional magnetic resonance imaging (rs-fMRI) can detect spontaneous brain functional activity, probe the pathogenesis of VCI in-depth and provide a reference for early diagnosis and prognostic evaluation.

Shen et al. demonstrated that methylene blue (MB) treatment can reverse mitochondrial dysfunction following oxygen glucose deprivation/reoxygenation (OGD) in PC12 cells, reduce neuronal apoptosis and improve blood-brain barrier (BBB) integrity in a fluid percussion TBI mouse model. MB also inhibited ROS production, stabilized neuronal mitochondrial membrane potential (MMP), increased ATP production, and preserved brain functions following TBI as demonstrated by Morris water maze, rotarod, and modified Neurological Severity Score (mNSS) tests.

Reduced cerebral blood flow (CBF) and perfusion may underlie persistent post-traumatic symptoms of mild-moderate TBI, but these parameters might be difficult to measure. Quinn et al. applied pseudo-continuous arterial spin labeling (pCASL) magnetic resonance imaging techniques in measuring CBF in 24 subjects with mild and moderate TBI before and after 10 days of computerized executive function training (CEFT) combined with active or sham anodal transcranial direct current stimulation (tDCS). Robust improvements in depression, anxiety, attention, and executive function were observed in both active and sham tDCS groups. tDCS stimulation was associated with static/increased CBF in the right inferior frontal gyrus whereas sham tDCS reduced CBF. Neuropsychological performance and behavioral symptoms, however, were not associated with changes in CBF, probably due to the lack of a control for CEFT. Thus, cerebral perfusion measured with MRI presents a potential pathophysiological target for rehabilitation paradigms of TBI.

Pape et al. showed in a randomized controlled trial that familiar auditory sensory training (a passive auditory stimulation delivered by means of recordings of autobiographical stories narrated by patients' relatives) facilitated changes in neural connectivity in patients with TBI-induced disorder of consciousness. They highlighted a relation between language-related neural networks and improvement in awareness.

In a randomized control trial and by using MoCA, STT, and Functional Activities Questionnaire (FAQ), Zhang et al. showed that playing mahjong for 12 weeks improved the executive function of elderly (74.3 ± 4.3) with mild cognitive impairment (MCI). Since mahjong is a popular 4-people game, further study should determine if mahjong can improve cognitive deficits in people with mild TBI.

Up to 50% of TBI patients suffer from insomnia which may underlie chronic inflammation and is unlikely to subside even after symptoms of mTBI remit. Dietch and Furst provide evidence supporting effectiveness of cognitive-behavioral therapy for insomnia (CBT-I) (≥50% clinical remission rate in general population) and propose more CBT-I studies in patients with mTBI because insomnia is a highly treatable symptom of mTBI that could have broad positive impacts on the recovery of TBI symptoms including cognitive deficits.

Photobiomodulation (PBM) is a therapy that uses red-to-near-infrared (NIR) light to heal injured tissue. Chao et al. reported that 8 weeks of PBM intervention [810-nm light-emitting diodes at 10 or 40 Hz by an intranasal and four transcranial modules targeting nodes of the default mode network (DMN)] increased brain volumes and cerebral perfusion, improved functional connectivity and neuropsychological test scores in a professional hockey player with a history of concussions and chronic symptoms of headaches, mild anxiety, and difficulty concentrating.

By meta-analysis of 36 randomized controlled trials, Lee et al. showed that herbal medicine (HM), more commonly used in China, Asian, and other developing countries as either monotherapy or as adjunctive therapy, are significantly better than conventional treatment (CT) on post-concussion syndrome (dizziness, headache, epilepsy, mental disorder, mild TBI-like symptoms), activities of daily living, neurological dysfunction, and safety profile. However, no differences in traumatic brain edema, posttraumatic hydrocephalus or cognitive dysfunction are found. As most HM studies reported were low-quality due to heterogeneity of the clinical populations, in diagnosis and in the use of active herbal ingredients for TBI treatment, further rigorous standardized RCTs of HB are needed.

As most TBI studies are done in wealthy countries with few data from developing countries, Adamson et al. in collaboration with Enhancing Neuro Imaging Genetics Through Meta-Analysis (ENIGMA) have emphasized the importance of international collaboration and domestic capacity building for innovative research, training clinicians and researchers and databases development to bring developing countries into the international platform for dementia and TBI research.

As we learn more about the evolving neuropathological processes and mechanisms underlying the post-traumatic syndromes following mTBI, better targeted interventions are more likely to be developed. The innovative work published in this Research Topic of Advances in Diagnosis and Treatment of TBI-Induced Neurodegeneration and Cognitive Deficits suggest that evidence-based anti-inflammatory, anti-hypoperfusion, and anti-anxiety/insomnia therapies show promise for TBI-induced post-traumatic symptoms and cognitive deficits.

## Author Contributions

All authors listed have made a substantial, direct and intellectual contribution to the work, and approved it for publication.

## Conflict of Interest

GX was employed by company Lotus Biotech.com LLC. The remaining authors declare that the research was conducted in the absence of any commercial or financial relationships that could be construed as a potential conflict of interest.

## Publisher's Note

All claims expressed in this article are solely those of the authors and do not necessarily represent those of their affiliated organizations, or those of the publisher, the editors and the reviewers. Any product that may be evaluated in this article, or claim that may be made by its manufacturer, is not guaranteed or endorsed by the publisher.

